# Is Laser Assisted Capsulotomy better than standard CCC?

**DOI:** 10.22336/rjo.2017.4

**Published:** 2017

**Authors:** Monica Gavriș, Radu Mateescu, Roxana Belicioiu, Ioana Olteanu

**Affiliations:** * Optisan Laser Clinic, Cluj-Napoca, Romania

**Keywords:** capsulorhexis, Utrata forceps, Femtosecond laser, white intumescent cataract

## Abstract

**Objectives:** To compare the safety and intraoperative difficulties of two capsulorhexis techniques for white intumescent cataract: Femtolaser-assisted capsulorhexis and manual capsulorhexis performed in 2-3 stages, with the Utrata forceps.

**Materials and methods:** A prospective comparative study that included 28 eyes divided into 2 equal groups in which capsulorhexis was performed by using the 2 methods. In the first group, the capsulorhexis was executed by using LenSx Femtolaser. In the second group, an Utrata forceps was used to perform a manual 2-3 steps capsulorhexis as follows: a small 2-3 mm capsulorhexis was performed after the staining of the anterior capsule with Trypan Blue along with a good pressurization with viscoelastic substance. The liquefied cortex was aspirated, followed by the enlargement of the capsulorhexis. In some cases, the enlargement was made after IOL implantation.

**Results:** In the Femtolaser group, the capsule was completely detached in 13 cases and only in one case, the capsule had a few bridges which detached easily, without endangering the capsulorhexis integrity. Its size was 4,9 mm in all cases. In the group in which capsulorhexis was performed with the Utrata forceps in 2-3 stages, this was complete, circular and relatively well centered in all cases, but the size varied between 4,5 and 5,5 mm.

**Conclusions:** Femtosecond laser-assisted capsulorhexis was round, well centered and of a desired size of 4,9 mm. The manual capsulorhexis with the Utrata forceps depends on the surgeon’s skill and experience and requires a good local anesthesia, the coloring of the anterior capsule with Tripan Blue, using a large quantity of cohesive viscoelastic substances and sometimes using micro incision forceps for helpful maneuvers. The size and centering of the capsulorhexis are not always identical with the intended ones.

## Introduction

Cataract surgery is one of the safest and most efficient surgical interventions, but it is extremely dependent on the surgeon’s experience, especially in challenging cases as intumescent cataract, subluxated lens cataract, hypermature cataract, cataract in the myopic or vitrectomized eye, etc.

Capsulorhexis is considered the most important step in cataract surgery and offers the lens bag more resistance during hydrodissection, phacoemulsification, and cortex aspiration, reducing the risk of capsular tears and improving the postoperative stability of the IOL. Capsulorhexis was first described in 1987 by Gimbel and Neuhann, as a circular, centered, curvilinear opening in the anterior capsule. These aspects help in maintaining a good positioning of the IOL in the capsular bag while decreasing the incidence of radial tears at the same time [**1**].

In a study by Gavris et al. in 2003, on 70 eyes with intumescent cataract, capsulorhexis skidding frequency was 11.43%, and in one case, 1.43% respectively, the posterior capsule rupture occurred [**2**].

These unfavorable results determined us to improve our method, by performing a 2-3 stages capsulorhexis as described by Gimbel et al. [**3**], or by implementing a new technological solution like Femtolaser-capsulotomy. Once Lasers were introduced in cataract surgery, a well-controlled and reproducible capsulorhexis was obtained, by laser-tissue interaction called photodisruption. In 2008, Zoltan Nagy performed the first laser capsulotomy during the first femtosecond laser-assisted cataract surgery [**4**].

## Objectives of the study

The objectives were to compare the safety and intraoperative difficulties of two capsulorhexis techniques for white intumescent cataract: the capsulorhexis performed with the LensEx femtosecond laser and the capsulorhexis performed in 2-3 stages with Utrata forceps.

## Materials and method

This is a prospective and comparative clinical study, which included a total of 28 patients (28 eyes) with white intumescent cataract.

The patients were operated on by the same surgeon at Optisan Laser Clinic, Cluj-Napoca, between July and December 2016. Patients were divided into 2 equal groups.

The first group was made up of 6 women and 8 men, aged between 20 and 73 years old, with an average age of 56,64 years and the second group was made up of 5 men and 9 women, aged between 57 and 78 years old, with an average age of 68,14 years.

A 4,9 mm Femtolaser capsulotomy was performed in the first group by using the LenSx laser, and in the second group, a 2-3 steps manual capsulorhexis was performed by using the Utrata forceps.

Surgery was performed under local anesthesia (Oxibuprocaine 0,4%) and pupil dilation was obtained with Tropicamide 1% and Neosynephrine 10%, one drop every 15 minutes, 60-90 minutes preoperatively.

**Fig. 1:**
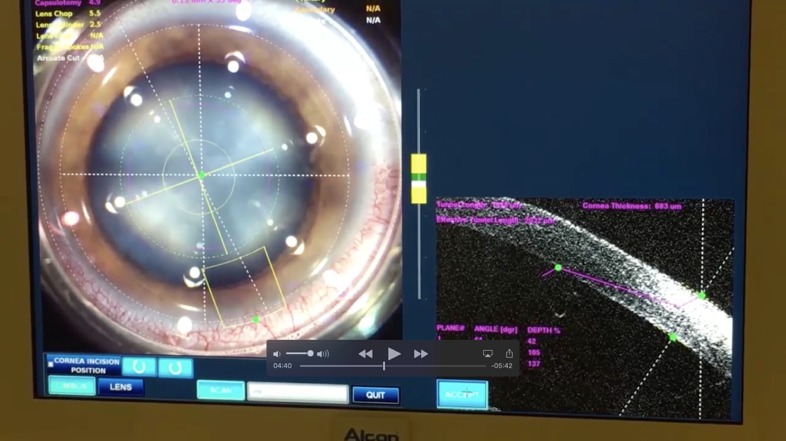
Surgical steps programming

For the first group, the first step was the Laser procedure and included:

Surgical steps programming (4,9 mm capsulorhexis and 2,2 mm incision) (**Fig. 1**).

**Fig. 2:**
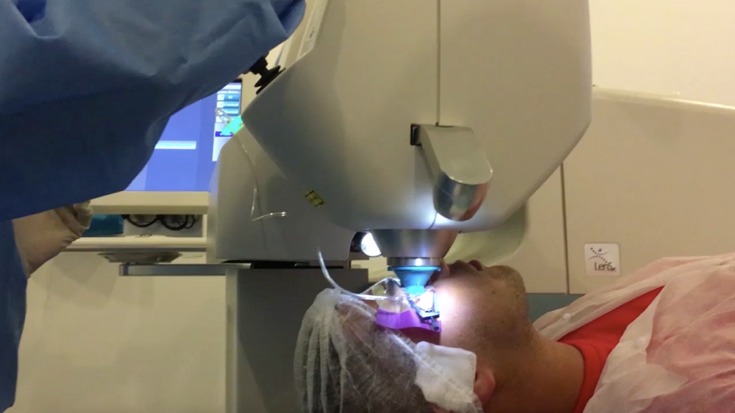
Performing the docking

**Fig. 3:**
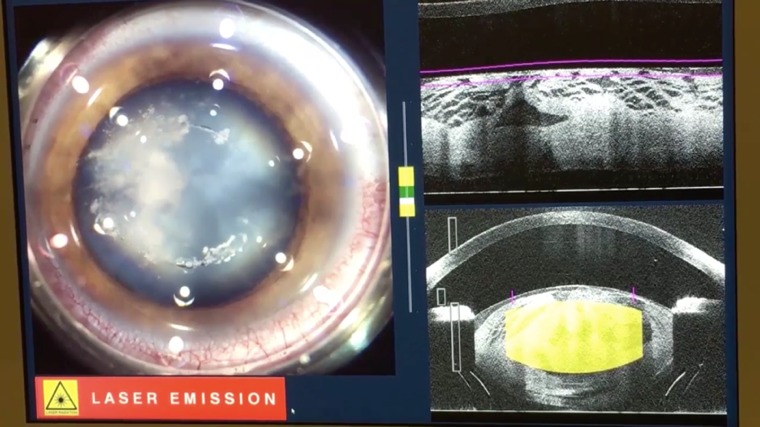
Centering the treatment plan and starting the Laser

- Performing the docking (**Fig. 2**).

- Centering the treatment plan and starting the Laser (**Fig. 3**).

The docking is the most important step in Femtolaser assisted cataract surgery and it determines the safety and accuracy of the entire procedure. Once it is properly done and the position of the eye is checked on the screen, suction is applied by simply pressing a button.

**Fig. 4:**
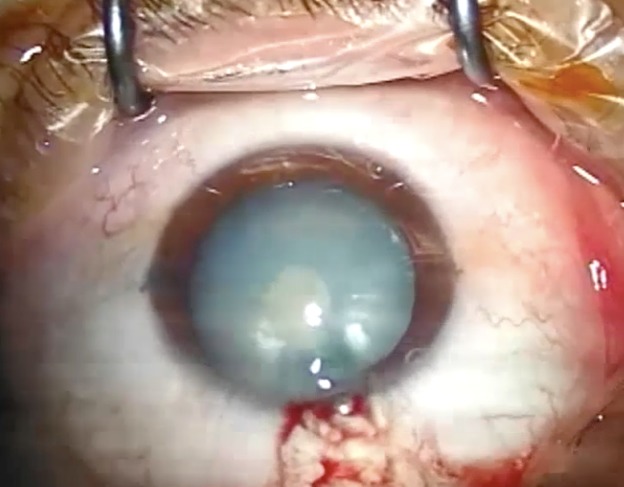
Creation a 2-3 m small, centered, round capsulorhexis

**Fig. 5:**
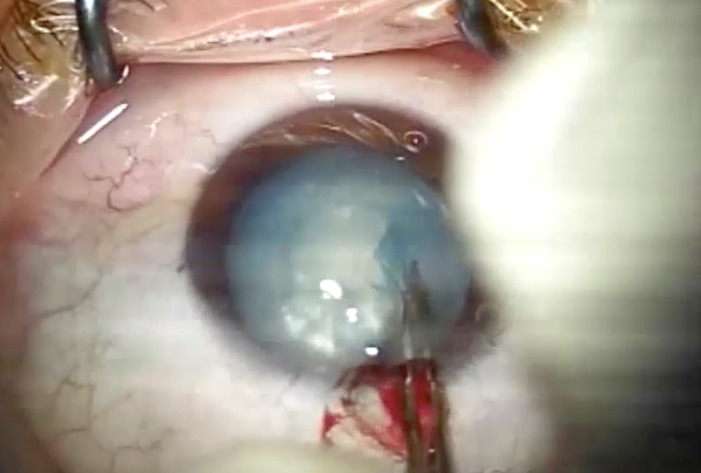
Widening of the capsulorhexis

In the second group, the capsulorhexis was performed in 2-3 stages by using the Utrata forceps, after the staining of the anterior capsule with Tripan Blue under an air bubble. Thus, after a good anterior capsule pressurization with viscoelastic substance, a 2-3 m small, centered, round capsulorhexis was performed (**Fig. 4**) and the liquefied cortex was aspirated, obtaining the decompression of the capsular bag. In some cases, the capsulorhexis widening was performed as the next step (**Fig. 5**) and in other cases, the widening was made after IOL implantation.

In the next stage, the same surgeon performed the phacoemulsification of the nucleus in both groups, by using the Centurion vision system, in the same operating room.

Pre- and postoperative patient evaluation was complex and included the determination of the visual acuity, measurement of the IOP, slit-lamp examination of the anterior segment, B-scan, ultrasound biometry, and endothelial cell counting. All the cases were evaluated the second day, at 1 week and at 6 months postoperatively.

No intra- or postoperative complications occurred. Postoperative topical therapy included topical antibiotics and steroidal anti-inflammatory drops for 6 weeks.

## Results

Preoperatively, there were no differences in the number of cases and disease staging in these 2 groups. Only the mean age was smaller in the group in which Femtosecond-capsulorhexis was performed (56,64 vs. 68,14).

Obtaining a curvilinear, continuous, intact capsulorhexis at the end of the surgery was considered a surgical success.

Out of 28 operated eyes, a Femtosecond capsulorhexis was performed on 14 eyes, and a 2-3 stages Utrata forceps capsulorhexis was performed on the other 14.

**Fig. 6:**
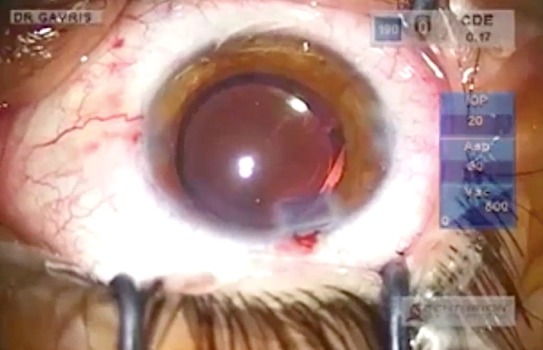
Capsule with a size of 4,9 mm

In the group in which the capsulorhexis was performed with the LenSx Laser, the capsule was completely detached in 13 cases (92,86%) (free-floating capsulotomy) and only in one case (7,14%), the capsule had a few bridges which detached easily, without endangering the capsulorhexis integrity. Its size was 4,9 mm in all cases (**Fig. 6**).

In the group in which capsulorhexis was performed with the Utrata forceps in 2-3 stages, this was complete, circular and relatively well centered in all cases, but the size varied between 4,5 and 5,5 mm. Several maneuvers for capsular bag decompression were made to insure the optimal size of the rhexis, and a lot of cohesive viscoelastic substance was used.

## Discussions

Peer-review studies have already demonstrated that the Femtolaser capsulotomy is better centered and more precise compared to manual capsulorhexis. A 360-degree overlapping capsular edge was thought to be an important feature for standardizing refractive results, preventing the optic decentration, shifts toward myopia or hyperopia, tilt or capsular opacification due to symmetric contractile forces of the capsular bag [**5**].

While performing the 1-stage capsulorhexis in white intumescent cataracts, the surgeon perceives the high intracapsular pressure and the leakage of the liquefied cortex, which can lead to discontinuities or tearing of the anterior or posterior capsule and difficulties in centering the capsulorhexis.

Newton Kara-Junior et al. compared the results of the 1-stage versus the 2-stage capsulorhexis in intumescent white cataracts and found anterior capsule tears in 23,07% of the cases in which the 1-stage capsulorhexis was performed (11 cases) and no ruptures of the anterior capsule were evidenced in which capsulorhexis was done in 2 stages (13 cases) [**6**].

In 1991, Gimbel analyzed 2967 cataract cases, out of which 34 were intumescent white cataracts and performed a 2-stages capsulorhexis in these cases. He found that in 4 out of the 34 cases, the anterior capsule tore [**7**].

The very good results we obtained by performing Femtosecond laser-capsulorhexis, as well as by a 2-3 stages capsulorhexis are owed to the surgeon’s experience and her concern in perfecting the capsulorhexis technique in intumescent white cataract, using the proper microsurgery instruments, and implementing new technological solutions [**8**].

## Conclusions

The achieved results confirmed the safety and efficacy of both techniques in performing the capsulorhexis in intumescent white cataracts.

The manual capsulorhexis with the Utrata forceps depends on the surgeon’s skill and experience and requires good local anesthesia, coloring of the anterior capsule with Trypan Blue, using a large quantity of cohesive viscoelastic substances, and sometimes using micro incision forceps for helpful maneuvers. Nevertheless, the size and centering of the capsulorhexis are not always identical with the intended ones.

Femtosecond laser-assisted capsulorhexis was round, well centered and of the desired size, 4,9 mm respectively.

The perfection of the Femtosecond laser-assisted capsulorhexis, along with the surgeon’s increased comfort, make this type of capsulorhexis a superior option or the technique of choice in intumescent white cataract cases, in which the risk of capsulorhexis skidding is greater than in other types of cataract.
